# Immune-related adverse events in older adults receiving immune checkpoint inhibitors: a comprehensive analysis of the Food and Drug Administration Adverse Event Reporting System

**DOI:** 10.1093/ageing/afaf008

**Published:** 2025-01-30

**Authors:** Buğra Han Esen, Sevval Nur Bektas, Umur Topcu, Bahadır Köylü, Fadime Buket Bayram Kuvvet, Gulistan Bahat, Fatih Selçukbiricik

**Affiliations:** School of Medicine, Koç University, Rumelifeneri Yolu 34450 Sarıyer, Istanbul, Turkey; School of Medicine, Koç University, Rumelifeneri Yolu 34450 Sarıyer, Istanbul, Turkey; School of Medicine, Koç University, Rumelifeneri Yolu 34450 Sarıyer, Istanbul, Turkey; Department of Medical Oncology, Koç University Hospital, Davutpaşa Street 34010 Topkapı, Istanbul, Turkey; Department of Internal Medicine, Koç University Hospital, Davutpaşa Street 34010 Topkapı, Istanbul, Turkey; Division of Geriatrics, Department of Internal Medicine, Istanbul Medical Faculty, Istanbul University, 34093 Fatih, Istanbul, Turkey; Department of Medical Oncology, Koç University Hospital, Davutpaşa Street 34010 Topkapı, Istanbul, Turkey

**Keywords:** immune checkpoint inhibitors, older adults, adverse event, pharmacovigilance, Food and Drug Administration Adverse Event Reporting System (FAERS), older people

## Abstract

**Background:**

Immune checkpoint inhibitors (ICIs) have revolutionised cancer therapy, yet they carry a unique spectrum of immune-related adverse events (irAEs). Given the ageing global population and the underrepresentation of older adults in clinical trials for ICIs, we investigated the occurrence and characteristics of irAEs in older versus younger adults as well as among different age subsets within the older adult population.

**Methods:**

We analysed the U.S. Food and Drug Administration Adverse Event Reporting System database reports from 2015 to 2023, focusing on ICIs. We categorised irAEs into 11 distinct types and performed descriptive and multivariate analyses to compare the prevalence and clinical characteristics of irAEs across different age groups, adjusting for potential confounding factors.

**Results:**

Among 47 513 patients aged 18–100 reporting irAEs, the 65–74 and 75–84 age groups had significantly increased risks compared to 18–64 (OR 1.13, 95% CI [1.09–1.18]; 1.15 [1.1–1.21]). Cardiovascular irAEs rose with age, peaking at 75–84, while endocrine irAEs decreased. Hepatobiliary, gastrointestinal and ocular irAEs decreased with age, but renal and musculoskeletal irAEs increased, showing higher risks in older adults. Serious outcomes slightly decreased in the 85+ group, while the proportion of deaths increased with age.

**Conclusion:**

We discuss the potential changes in the immune system contributing to the decreased prevalence of irAEs in the oldest age group. Additionally, conservative treatment approaches and underreporting of irAEs in older patients may influence these findings. Our findings highlight the need for personalised decision-making for ICI therapies, considering performance status and comorbidities rather than age alone.

## Key Points

- The 85+ group shows decreased overall immune-related adverse event (irAE) risk compared to 65–74 and 75–84.
- The 65–74 and 75–84 groups have increased overall irAE risk compared to the 18–64 group.
- For cardiovascular irAEs, older patients exhibit increased toxicity risk.
- Hepatobiliary-related and endocrine-related adverse events show a lower risk in younger adults compared to older adults.
- No significant toxicity changes were found for immune-related cutaneous, neurological and haematological events across age group.

## Introduction

Immune checkpoint inhibitors (ICIs), targeting inhibitory receptors such as Programmed cell death protein 1 (PD-1), Programmed death-ligand 1 (PD-L1) and Cytotoxic T-lymphocyte-associated protein 4 (CTLA-4), have revolutionised cancer therapy [1]. These monoclonal antibodies enhance T-cell responses against tumour cells, establishing themselves as first-line treatments for various cancers [2]. ICIs’ notable efficacy and safety profile have led to their broad adaption, benefitting a wide variety of patients including older adults [3]. This shift in oncology underscores the significant role of ICIs in broadening treatment options and achieving sustained clinical outcomes.

While ICIs are effective and relatively tolerable, they are associated with a unique spectrum of immune-related adverse events (irAEs), affecting multiple organ systems [4, 5]. These irAEs ranging from gastrointestinal disturbances to cutaneous reactions and endocrine dysfunctions may necessitate hospitalisation or treatment discontinuation and can be life-threatening, and a multidisciplinary approach to management with early detection is critical [5, 6]. Older adults are often underrepresented in ICI clinical trials. The efficacy and safety of immunotherapy constitute a gap in the literature. The global rise in the ageing population also necessitates a deeper understanding of ICIs in geriatrics, especially considering immunosenescence-related changes [7]. Immunosenescence, which is characterised by increased responsiveness to tolerogenic signals, is the age-associated remodelling of the immune system [8]. This process could potentially affect the severity, characteristics and frequency of irAEs in the older population.

Given the limited data available from clinical trials in older patients, it is crucial to understand the impact of ICIs in this underrepresented demographic. In this study, we investigated the occurrence of irAEs in older adults utilizing the U.S. Food and Drug Administration Adverse Event Reporting System (FAERS), the largest publicly available repository of unsolicited reports. Our objectives were to compare the occurrence and characteristics of irAEs in older versus younger adult patients. Further analyses looking into the differences among age-specific subsets and irAEs affecting different organ systems were done. By leveraging FAERS data, this analysis aimed to contribute to evidence-based medicine by aiding decision-making processes regarding the initiation and continuation of ICI therapy in older adults.

## Methods

### Data source

The U.S. Food and Drug Administration Adverse Event Reporting System (FAERS) is a comprehensive database that supports U.S. Food and Drug Administration's (FDA) postmarketing safety surveillance program for marketed drug and therapeutic biologic products. This database contains detailed information about adverse events, medication errors and product quality complaints that result in adverse events. The FAERS database receives voluntary submissions from including physicians, pharmacists, nurses and dentists and consumers who can report directly to the FDA, as well as mandatory submissions from manufacturers who are required by regulation to report adverse events through various channels including clinical trials, spontaneous reports from healthcare settings (hospitals, clinics, pharmacies), postmarketing surveillance studies and patient support programs. The database is structured into seven distinct datasets that capture various aspects of each report: patient demographics and administrative information (DEMO), drug details (DRUG), adverse event coding (REAC), patient outcomes (OUTC), report sources (RPSR), therapy start and end dates (THER) and indications for drug use (INDI). More information about FAERS can be found at https://fis.fda.gov/extensions/FPD-FAQ/FPD-FAQ.html#_Toc514144622. Our examination was concentrated on ICIs that received FDA approval up to 30 September 2023. The ICIs analysed included anti-PD-1 agents (nivolumab, pembrolizumab, cemiplimab, dostarlimab, retifanlimab), anti-PD-L1 agents (atezolizumab, avelumab, durvalumab), anti-CTLA-4 agents (ipilimumab, tremelimumab) and an anti-LAG-3 agent (relatlimab). These agents were targeted using specific keywords to extract relevant case reports for the period between 1 January 2015 and 30 September 2023, from the FAERS database.

Adverse event reporting within the FAERS database employs preferred term (PT) codes from the Medical Dictionary for Regulatory Activities (MedDRA), which uniquely catalogues symptoms and adverse reactions [9]. For the definition of irAEs, we referred to AE terms found in irAE management guidelines and clinical reviews [10–16]. Further, irAEs were categorised into 11 distinct types: cardiovascular, endocrine, gastrointestinal, haematologic, hepatobiliary, pulmonary, musculoskeletal, neurological, ophthalmological, renal and cutaneous toxicities. The PTs associated with these irAE categories are documented in Table S1 in the appendix. Following National Institutes of Health criteria, we segmented the patient population into two primary age groups: young adults (under 65 years) and older adults (65 years and above) [17]. To enable more comprehensive comparisons across geriatric populations and align with established geriatric oncology literature focusing on irAEs, we further stratified the older adult cohort into age groups of 65–74 years, 75–84 years and 85 years and older. [18, 19] The outcomes were categorised as ‘serious’ and ‘nonserious’ adverse events based on the standardised outcome criteria in the FAERS database. Serious adverse events were defined as reports containing one or more of the following outcomes: death, life-threatening conditions, hospitalisation, disability, congenital anomaly, required intervention to prevent permanent impairment or other serious medical events. Reports that did not include any of these were classified as ‘nonserious’. The exact cause of these outcomes, whether related to the underlying cancer or treatment toxicity, was not established.

### Data processing procedure

Initially, we retrieved 14 109 895 case reports from the FAERS database. Following a deduplication process in line with FDA guidelines—which involved selecting the most recent FDA_DT for cases with identical CASEIDs and choosing the higher PRIMARYID when both identifiers were the same—we were left with 12 216 999 unique case reports for detailed examination. For inclusion in our analysis, cases needed to have ICIs listed as the ‘primary suspect’ in the ROLE_COD section. Cases were classified as monotherapy if a report indicated a single ICI as the ‘primary suspect’. Conversely, polytherapy cases involved the use of multiple ICIs, with at least one marked as ‘primary suspect’ and the others as ‘second suspect’, ‘concomitant’ or ‘interacting’. Similarly, chemotherapy and targeted therapy combinations were identified when these agents were reported in the ‘second suspect’, ‘concomitant’ or ‘interacting’ sections of the reports.

From this refined dataset, 138 195 cases were identified where an ICI was the primary suspect. We further excluded cases involving individuals younger than 18 or older than 100 years old, or where age information was unavailable, resulting in 90 990 cases. The International Nonproprietary Names for chemotherapies, targeted therapies and immunotherapies analysed in this study were obtained from the WHO Collaborating Centre for Drug Statistics Methodology under the classification ‘L01 ANTINEOPLASTIC AGENTS’. The exhaustive lists of these medications are provided in Tables S2 and S3 in the appendix [20].

### Statistical analysis

Our initial descriptive analysis included key demographic and clinical information such as sex, age, clinical outcomes, reporter type, ICI regimen, medical indications and any additional treatments. Countries were classified by income levels, detailed in Table S4 in the appendix.

To analyse the differences in toxicity profiles across age groups, we utilised multivariate analysis, incorporating strategies from previous studies that examined demographic impacts on irAEs [21, 22]. We identified and adjusted for potential confounding factors that are known to influence the likelihood of irAEs, based on insights from existing literature. These factors encompassed the specific ICI regimen [23], any additional chemotherapy or targeted therapy [23–25], medical indications for treatment [26], patient sex [22], the year of report submission [27, 28], the economic status of the reporting country [27, 28] and whether a healthcare professional filed the report [29]. The individual significance of these variables in relation to overall irAE development was assessed through univariate logistic regression analyses (Table S5 in the appendix). The statistical analyses were conducted using R software, version 4.3.1, with a predetermined significance level of *P* < .05. The data supporting our analysis can be accessed at https://fis.fda.gov/extensions/FPD-QDE-FAERS/FPD-QDE-FAERS.html.

### Ethical considerations

Given the FAERS database’s status as a publicly available and anonymised dataset, our study did not necessitate ethical approval or informed consent.

## Results

In our examination of the FAERS database, out of 90 990 cases using ICI therapy, 47 513 patients aged 18–100 reported irAEs. Of these, 42.1% (20 021) were aged 18–64, 36.2% (17 225) were 65–74, 19.2% (9138) were 75–84 and 2.4% (1129) were aged over 85. Demographic and clinical characteristics are detailed in Table 1.

**Table 1:** Demographics, clinical characteristics and age-based differences in patients with AEs following ICI administration reported in the FAERS database

Clinical characteristics	Patients aged 18–64 (%)		Patients aged 65–74 (%)		Patients aged 75–84 (%)		Patients aged 85+ (%)	
	irAE cases *n* = 20,021	Non- irAE cases *n* = 19,933	irAE cases *n* = 17,225	Non- irAE cases *n* = 14,718	irAE cases *n* = 9138	Non- irAE cases *n* = 7666	irAE cases *n* = 1129	Non- irAE cases *n* = 1160
**Sex**								
Male	11 374 (57.37%)	11 414 (58.56%)	11 570 (67.95%)	9769 (67.96%)	6124 (68.01%)	5065 (68.00%)	716 (64.57%)	713 (64.56%)
Female	8451 (42.63%)	8076 (41.44%)	5464 (32.05%)	4606 (32.04%)	2882 (31.99%)	2391 (32.00%)	393 (35.43%)	403 (35.44%)
Missing	196	443	191	343	132	210	20	44
**Age (Median, q1–q3, % data included)**	(56, 50–61, 100%)	(56, 49–61 (100%)	(70, 67–72, 100%)	(69, 67–72 (100%)	(78, 76–80, 100%)	(78, 76–80 (100%)	(86, 85–88, 100%)	(87, 85–89) (100%)
**Time to onset (Median, q1–q3, % data included)**	(41, 14–105) (57.99%)	(44, 14–124) (52.74%)	(42, 14–108) (59.64%)	(46, 14–129 (54.48%)	(41, 14–107) (58.35%)	(48, 14–131 (51.41%)	(44.5, 18–123.5) (50.84%)	(43, 11–144.25 (40.34%)
**Reporter type**								
Healthcare worker	16 896 (84.83%)	14 455 (73.52%)	14 297 (83.67%)	10 781 (74.28%)	7536 (83.23%)	5658 (75.11%)	914 (81.39%)	786 (68.59%)
Consumer	3019 (15.17%)	5205 (26.48%)	2812 (16.77%)	3733 (25.72%)	1526 (16.77%)	1875 (24.89%)	209 (18.61%)	360 (31.41%)
Missing	106	273	116	204	76	133	6	14
**Income level by country**								
High income	17 111 (91.95%)	15 167 (84.76%)	15 226 (94.73%)	11 755 (89.46%)	8267 (96.59%)	6446 (92.98%)	1057 (98.14%)	1021 (94.28%)
Upper middle income	1382 (7.43%)	1969 (11.00%)	776 (4.83%)	1084 (8.25%)	266 (3.11%)	388 (5.60%)	20 (1.86%)	51 (4.71%)
Lower to middle income	115 (0.62%)	758 (4.24%)	71 (0.44%)	301 (2.29%)	26 (0.30%)	99 (1.43%)	0 (0%)	11 (1.02%)
Missing	1413	2039	1152	1578	579	733	52	77
**Received year**								
2023	2738 (13.67%)	2338 (11.73%)	2449 (14.22%)	1758 (11.94%)	1354 (14.82%)	991 (12.94%)	160 (14.17%)	144 (12.41%)
2022	3085 (15.40%)	3355 (16.83%)	2876 (16.7%)	2548 (17.31%)	1558 (17.05%)	1340 (17.49%)	189 (16.74%)	220 (18.97%)
2021	2977 (14.86%)	2880 (14.45%)	2676 (15.54%)	2199 (14.94%)	1454 (15.91%)	1160 (15.13%)	174 (15.41%)	163 (14.05%)
2020	2610 (13.03%)	2411 (12.1%)	2258 (13.11%)	1814 (12.33%)	1202 (13.15%)	962 (12.55%)	159 (14.08%)	161 (13.88%)
2019	2840 (14.18%)	2683 (13.46%)	2479 (14.39%)	2031 (13.8%)	1299 (14.22%)	1130 (14.74%)	150 (13.29%)	154 (13.28%)
2018	2438 (12.17%)	2406 (12.07%)	2074 (12.04%)	1811 (12.3%)	1049 (11.48%)	921 (12.02%)	149 (13.2%)	133 (11.47%)
2017	1632 (8.15%)	1778 (8.92%)	1267 (7.36%)	1242 (8.44%)	648 (7.09%)	577 (7.54%)	73 (6.47%)	91 (7.84%)
2016	1013 (5.06%)	1260 (6.32%)	676 (3.92%)	862 (5.86%)	371 (4.06%)	408 (5.32%)	48 (4.25%)	74 (6.38%)
2015	687 (3.43%)	820 (4.12%)	464 (2.69%)	452 (3.08%)	202 (2.21%)	173 (2.27%)	27 (2.39%)	20 (1.72%)
Missing	1	2	6	1	1	4	0	0
**Indication organ/system**								
Head and neck	589 (3.07%)	997 (5.46%)	441 (2.67%)	568 (4.19%)	211 (2.41%)	239 (3.39%)	26 (2.43%)	43 (3.22%)
Lung	5770 (30.07%)	5511 (30.20%)	6722 (40.66%)	5530 (40.83%)	3236 (37.03%)	2596 (36.88%)	287 (26.85%)	299 (29.34%)
Urogenital	2702 (14.08%)	2308 (12.65%)	2831 (17.12%)	2101 (15.51%)	1614 (18.43%)	1248 (17.73%)	197 (18.43%)	169 (16.58%)
Gynecologic	798 (4.16%)	757 (4.15%)	478 (2.89%)	392 (2.89%)	168 (1.92%)	173 (2.46%)	7 (0.65%)	16 (1.57%)
Breast	965 (5.03%)	799 (4.38%)	251 (1.52%)	170 (1.26%)	80 (0.92%)	71 (1.01%)	7 (0.65%)	6 (0.59%)
Haematopoietic and lymphoid	455 (2.37%)	522 (2.86%)	242 (1.46%)	261 (1.93%)	133 (1.52%)	165 (2.34%)	18 (1.68%)	26 (2.55%)
Gastrointestinal	1143 (5.96%)	1614 (8.84%)	979 (5.92%)	1031 (7.61%)	528 (6.04%)	514 (7.3%)	60 (5.61%)	63 (6.18%)
Other indications	682 (3.55%)	801 (4.39%)	441 (2.67%)	428 (3.16%)	207 (2.37%)	209 (2.97%)	26 (2.43%)	35 (3.43%)
Hepatopancreatobiliary	759 (3.96%)	1044 (5.72%)	735 (4.45%)	851 (6.28%)	435 (4.98%)	444 (6.31%)	69 (6.45%)	71 (6.97%)
Skin	4917 (25.63%)	3057 (16.75%)	3082 (18.64%)	1738 (12.83%)	1904 (21.79%)	1128 (16.02%)	352 (32.93%)	244 (23.95%)
Other thoracic (cardiac, mesothelioma, thymus)	123 (0.64%)	159 (0.87%)	223 (1.35%)	212 (1.57%)	171 (1.96%)	160 (2.27%)	13 (1.22%)	21 (2.06%)
Musculoskeletal	120 (0.63%)	259 (1.42%)	45 (0.27%)	101 (0.75%)	32 (0.37%)	42 (0.60%)	4 (0.37%)	16 (1.57%)
CNS	110 (0.57%)	318 (1.74%)	32 (0.19%)	110 (0.81%)	6 (0.07%)	31 (0.44%)	1 (0.09%)	2 (0.20%)
Endocrine	54 (0.28%)	103 (0.56%)	32 (0.19%)	51 (0.38%)	13 (0.15%)	20 (0.28%)	2 (0.19%)	8 (0.79%)
Unspecified or missing	834	1684	691	1174	400	626	60	141
**Dechallenge**								
Applied	7605 (81.13%)	3661 (43.77%)	7067 (79.51%)	3275 (50.24%)	3715 (77.48%)	1661 (47.88%)	436 (73.90%)	200 (36.63%)
Positive	6457 (84.90%)	2907 (79.40%)	6001 (84.92%)	2550 (77.86%)	3118 (83.93%)	1279 (77.00%)	357 (81.88%)	146 (73.00%)
Negative	1148 (15.10%)	754 (20.60%)	1066 (15.08%)	725 (22.14%)	597 (16.07%)	382 (23.00%)	79 (18.12%)	54 (27.00%)
Not applied	1748 (18.87%)	4705 (56.23%)	1820 (20.49%)	3245 (49.76%)	1079 (22.52%)	1808 (52.12%)	154 (26.1%)	346 (63.37%)
Missing	10 668	11 567	8338	8198	4344	4197	539	614
**Rechallenge**								
Applied	338 (33.67%)	233 (24.84%)	310 (34.83%)	187 (31.22%)	148 (36.36%)	95 (30.84%)	16 (22.54%)	17 (34.00%)
Positive	131 (38.76%)	75 (32.19%)	115 (37.10%)	68 (36.36%)	50 (33.78%)	29 (33.78%)	7 (43.75%)	4 (23.53%)
Negative	207 (61.24%)	158 (67.81%)	195 (62.90%)	119 (63.64%)	98 (66.22%)	66 (66.22%)	9 (56.25%)	13 (76.47%)
Not applied	666 (66.33%)	705 (75.16%)	580 (65.17%)	412 (68.78%)	259 (63.64%)	213 (69.16%)	55 (77.46%)	33 (66.00%)
Missing	19 017	18 995	16 335	14 119	8731	7358	1058	1110
**Outcomes**								
Nonserious outcome	794 (3.97%)	1739 (8.72%)	658 (3.82%)	1089 (7.4%)	383 (4.19%)	637 (8.31%)	89 (7.88%)	174 (15%)
Serious outcome	19 227 (96.03%)	18 194 (91.28%)	16 567 (96.18%)	13 629 (92.6%)	8755 (95.81%)	7029 (91.69%)	1040 (92.12%)	986 (85%)
Death	2951 (14.74%)	6768 (33.95%)	3202 (18.59%)	5075 (34.48%)	2105 (23.04%)	2743 (35.78%)	249 (22.05%)	466 (40.17%)
Hospitalisation	11 649 (58.18%)	8471 (42.5%)	10 378 (60.25%)	6577 (44.69%)	5473 (59.89%)	3236 (42.21%)	613 (54.3%)	374 (32.24%)
Life-threatening	2039 (10.18%)	1001 (5.02%)	1926 (11.18%)	771 (5.24%)	1041 (11.39%)	395 (5.15%)	97 (8.59%)	55 (4.74%)
Disability	632 (3.16%)	328 (1.65%)	584 (3.39%)	293 (1.99%)	359 (3.93%)	147 (1.92%)	40 (3.54%)	20 (1.72%)
Required intervention to prevent permanent impairment/damage	35 (0.17%)	10 (0.05%)	48 (0.28%)	14 (0.1%)	31 (0.34%)	9 (0.12%)	5 (0.44%)	2 (0.17%)
Other serious	15 095 (75.4%)	12 210 (61.26%)	13 004 (75.49%)	8952 (60.82%)	6924 (75.77%)	4678 (61.02%)	806 (71.39%)	695 (59.91%)
**Dosage (Median, q1–q3) (% data included)**								
Cumulative total dosage	(662.58, 222.6–1781.61) (12.34%)	(720, 240–2280 (8.89%)	(600, 200–1600) (15%)	(720, 240–2160 (10.91%)	(600, 200–1500) (15.08%)	(600, 216–1800 (10.66%)	(400, 200–1500) (13.91%)	(830, 240–1710 (8.62%)
Cumulative total dosage, per kg	(10, 3.51–25) (9.9%)	(9.99, 3.7–27) (6.56%)	(9.38, 3.7–25) (12.23%)	[4–30] (7.74%)	(9.8, 3.98–23.53) (11.85%)	(9.84, 3.92–24.89) (7.77%)	(7.41, 3.92–26.44) (9.65%)	(11.94, 5–30) (5.6%)
Total dosage, per shot	(240, 200–710) (55.48%)	(240 200–740) (59.9%)	(240 200–560) (59.58%)	(240 200–840) (60.32%)	(225 200–480) (58.56%)	(240 200–615) (59.31%)	(200 200–400) (55.71%)	(240, 200–480) (54.31%)
Total dosage, per shot, per kg	(3, 2.86–9.16) (45.95%)	(3.08, 3–10) (37.95%)	(3.2, 2.99–9.77) (46.8%)	(3.45, 3–10) (39.4%)	(3.29, 3–9.23) (44.97%)	(3.8, 3–10 (36.15%)	(3.12, 2.84–6) (39.06%)	(3.57, 2.99–10 (27.84%)
**Treatment regimen**								
No additional chemotherapy	16 126 (80.55%)	17 131 (85.94%)	13 833 (80.31%)	12 542 (85.22%)	7838 (85.77%)	6807 (88.79%)	1052 (93.18%)	1102 (95%)
Additional chemotherapy	3895 (19.45%)	2802 (14.06%)	3392 (19.69%)	2176 (14.78%)	1300 (14.23%)	859 (11.21%)	77 (6.82%)	58 (5%)
No additional targeted therapy	16 923 (84.53%)	17 420 (87.39%)	14 630 (84.93%)	12 934 (87.88%)	8002 (87.57%)	6821 (88.98%)	1018 (90.17%)	1047 (90.26%)
Additional targeted therapy	3098 (15.47%)	2513 (12.61%)	2595 (15.07%)	1784 (12.12%)	1136 (12.43%)	845 (11.02%)	111 (9.83%)	113 (9.74%)
No polytherapy	15 040 (75.12%)	16 536 (82.96%)	13 680 (79.42%)	12 441 (84.53%)	7542 (82.53%)	6659 (86.86%)	1004 (88.93%)	1028 (88.62%)
Polytherapy	4981 (24.88%)	3397 (17.04%)	3545 (20.58%)	2277 (15.47%)	1596 (17.47%)	1007 (13.14%)	125 (11.07%)	132 (11.38%)
**Number of different immunotherapy agents used**								
**Monotherapy**	15 040 (75.12%)	16 536 (82.96%)	13 680 (79.42%)	12 441 (84.53%)	7542 (82.53%)	6659 (86.86%)	1004 (88.93%)	1028 (88.62%)
Anti-PD-1	10 581 (52.85%)	11 642 (58.41%)	9888 (57.4%)	8444 (57.37%)	5547 (60.7%)	4712 (61.47%)	796 (70.5%)	808 (69.66%)
Nivolumab	5650 (28.22%)	7445 (37.35%)	5116 (29.7%)	5222 (35.48%)	2705 (29.6%)	2637 (34.4%)	330 (29.23%)	419 (36.12%)
Pembrolizumab	4859 (24.27%)	4116 (20.65%)	4700 (27.29%)	3151 (21.41%)	2794 (30.58%)	2038 (26.58%)	441 (39.06%)	360 (31.03%)
Cemiplimab	52 (0.26%)	49 (0.25%)	57 (0.33%)	43 (0.29%)	45 (0.49%)	34 (0.44%)	23 (2.04%)	29 (2.5%)
Dostarlimab	20 (0.1%)	32 (0.16%)	15 (0.09%)	28 (0.19%)	3 (0.03%)	3 (0.04%)	2 (0.18%)	0 (0%)
Anti-PD-L1	3551 (17.74%)	4322 (21.68%)	3251 (18.87%)	3667 (24.92%)	1709 (18.7%)	1788 (23.32%)	174 (15.41%)	203 (17.5%)
Atezolizumab	2653 (13.25%)	2999 (15.05%)	2289 (13.29%)	2315 (15.73%)	1161 (12.71%)	1075 (14.02%)	124 (10.98%)	122 (10.52%)
Durvalumab	715 (3.57%)	1063 (5.33%)	755 (4.38%)	1095 (7.44%)	409 (4.48%)	524 (6.84%)	23 (2.04%)	46 (3.97%)
Avelumab	183 (0.91%)	260 (1.3%)	207 (1.2%)	257 (1.75%)	139 (1.52%)	189 (2.47%)	27 (2.39%)	35 (3.02%)
Anti-CTLA-4	908 (4.54%)	572 (2.87%)	541 (3.14%)	330 (2.24%)	286 (3.13%)	159 (2.07%)	34 (3.01%)	17 (1.47%)
Ipilimumab	906 (4.53%)	569 (2.85%)	540 (3.13%)	329 (2.24%)	285 (3.12%)	159 (2.07%)	34 (3.01%)	17 (1.47%)
Tremelimumab	2 (0.01%)	3 (0.02%)	1 (0.01%)	1 (0.01%)	1 (0.01%)	0 (0%)	0 (0%)	0 (0%)
**Dual therapy**	4891 (24.43%)	3363 (16.87%)	3472 (20.16%)	2247 (15.27%)	1573 (17.21%)	989 (12.9%)	123 (10.89%)	131 (11.29%)
Nivolumab + ipilimumab	4441 (22.18%)	3023 (15.17%)	3113 (18.07%)	1978 (13.44%)	1411 (15.44%)	884 (11.53%)	115 (10.19%)	118 (10.17%)
Nivolumab + pembrolizumab	36 (0.18%)	15 (0.08%)	35 (0.2%)	16 (0.11%)	17 (0.19%)	6 (0.08%)	1 (0.09%)	2 (0.17%)
Pembrolizumab + ipilimumab	120 (0.6%)	53 (0.27%)	71 (0.41%)	16 (0.11%)	31 (0.34%)	13 (0.17%)	2 (0.18%)	2 (0.17%)
Nivolumab + relatlimab	35 (0.17%)	73 (0.37%)	39 (0.23%)	51 (0.35%)	23 (0.25%)	28 (0.37%)	2 (0.18%)	7 (0.6%)
Durvalumab + tremelimumab	228 (1.14%)	173 (0.87%)	192 (1.11%)	159 (1.08%)	78 (0.85%)	44 (0.57%)	3 (0.27%)	1 (0.09%)
**Triple therapy or more**	90 (0.45%)	30 (0.15%)	73 (0.42%)	30 (0.2%)	23 (0.25%)	18 (0.23%)	2 (0.18%)	1 (0.09%)
Nivolumab + pembrolizumab + atezolizumab	0 (0%)	14 (0.07%)	0 (0%)	21 (0.14%)	2 (0.02%)	5 (0.07%)	0 (0%)	1 (0.09%)
Nivolumab + ipilimumab + relatlimab	12 (0.06%)	9 (0.05%)	16 (0.09%)	5 (0.03%)	4 (0.04%)	12 (0.16%)	0 (0%)	0 (0%)
**Number of different chemotherapy agents used**								
None	16 126 (80.55%)	17 131 (85.94%)	13 833 (80.31%)	12 542 (85.22%)	7838 (85.77%)	6807 (88.79%)	1052 (93.18%)	1102 (95%)
One	875 (4.37%)	787 (3.95%)	682 (3.96%)	509 (3.46%)	287 (3.14%)	226 (2.95%)	28 (2.48%)	21 (1.81%)
Two or more	3020 (15.08%)	2015 (10.11%)	2710 (15.73%)	1667 (11.33%)	1013 (11.09%)	633 (8.26%)	49 (4.34%)	37 (3.19%)
**Number of different targeted therapy agents used**								
None	16 923 (84.53%)	17 420 (87.39%)	14 630 (84.93%)	12 934 (87.88%)	8002 (87.57%)	6821 (88.98%)	1018 (90.17%)	1047 (90.26%)
One	2632 (13.15%)	2228 (11.18%)	2389 (13.87%)	1645 (11.18%)	1053 (11.52%)	799 (10.42%)	105 (9.3%)	109 (9.4%)
Two or more	466 (2.33%)	285 (1.43%)	206 (1.2%)	139 (0.94%)	83 (0.91%)	46 (0.6%)	6 (0.53%)	4 (0.34%)

Sex-based differences were noted among age groups, with males comprising about twice the proportion of females in all three older age groups for both irAE and non-irAE cohorts. Lung cancer was the most common indication for ICI use in the 18–64, 65–74 and 75–84 age groups (30.1%, 40.7% and 37%, respectively). In contrast, for those over 85, skin cancer was the most frequent at 32.9%. Lung cancer remained a common indication in non-irAE cases as well (Table 1).

Among the treatment regimens, monotherapy was the predominant one across age groups, increasing with age among older patients with irAEs (75.1%, 79.4%, 82.5%, 88.9%) and slightly higher in non-irAE cohorts. Anti-PD-1 therapies also increased similarly but with differences in specific agent use; nivolumab was less common in irAE patients, while pembrolizumab was more prevalent. Anti-CTLA-4 usage remained low across all older groups. Dual therapy decreased with age in irAE groups (24.4%, 20.2%, 17.2%, 10.9%) and was even lower in non-irAE patient age groups with and without irAEs. Moreover, dual therapy use decreased with age in the irAE groups (24.4%, 20.2%, 17.2%, 10.9%). In all patients with non-irAEs, the same pattern was present with lower percentages (Table 1). Regarding the additional treatments, the data show a trend of decreasing additional chemotherapy and targeted therapies with advancing age. Non-irAE groups have consistently lower use of additional therapy in their regimens than irAE groups. When looking at outcomes, the frequency of serious outcomes modestly decreased in the 85+ age group in comparison to younger age groups with irAEs. Conversely, the proportion of deaths showed an increase with age in the irAE groups (Table 1).

In Figure S1 in the appendix, the incidence of overall irAEs is relatively consistent across ages. Some organ systems exhibit consistent trends across age groups. Further details of irAE rates in each organ system can be found in the figure.

**Figure 1:**
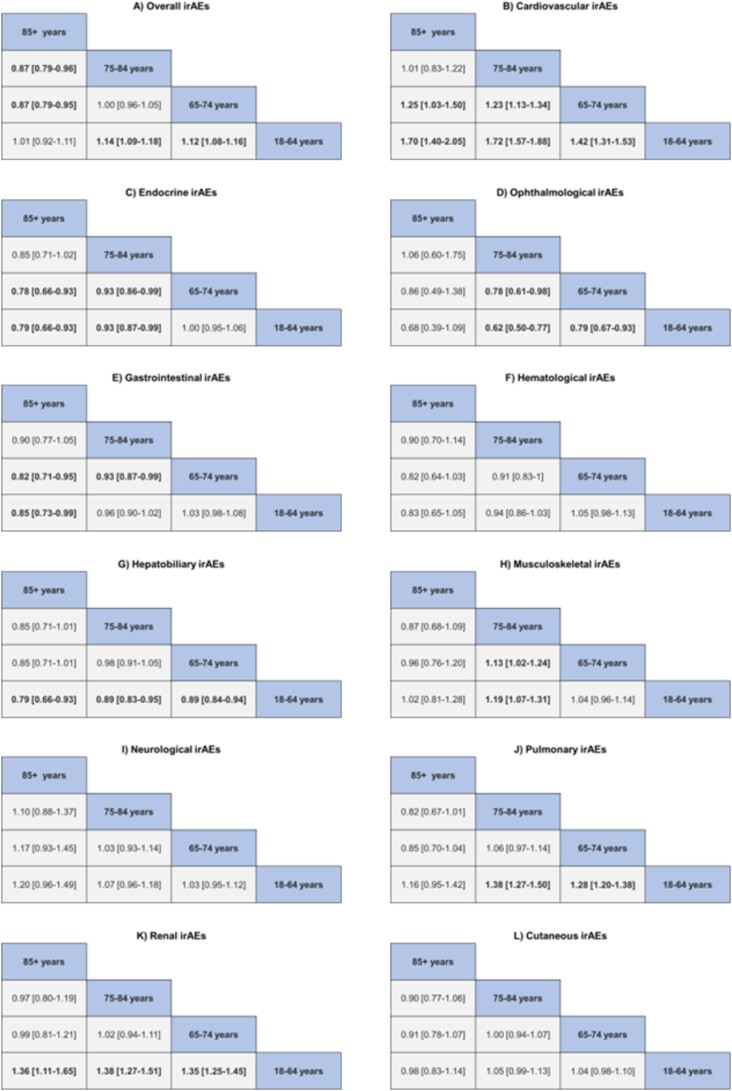
Multivariate regression analysis of irAE risk between age groups with adjusted ORs and confidence intervals

Figure 1 presents the adjusted odds ratios (ORs) and confidence intervals (CIs) from a multivariate regression analysis, comparing irAE risks across age groups. The data show increased irAE risks in the 65–74 and 75–84 age groups (1.12 [1.08–1.16], 1.14 [1.09–1.18]) relative to the 18–64 age group. However, the risk does not significantly change between the 18–64 age group and the 85+ age group, nor between the 65–74 and 75–84 age groups. The 85+ age group shows a decreased risk compared to the 65–74 and 75–84 age groups (0.87 [0.79–0.95], 0.87 [0.79–0.96]).

For cardiovascular irAEs, older patients show a clear trend of increased toxicity risk. Specifically, the 65–74 age group has a higher risk than the 18–64 age group (1.42 [1.31–1.53]), and the 75–84 age group has a higher risk than the 65–74 age group (1.23 [1.13–1.34]). For endocrine-related adverse events, the risk decreases with age. ORs indicate reduced risks for the 75–84 age group compared to the 18–64 and 65–74 age groups (0.93 [0.87–0.99] for both comparisons). The 85+ age group also shows lower risks compared to the 18–64 (0.79 [0.66–0.93]) and 65–74 (0.78 [0.66–0.93]) age groups. For hepatobiliary-related adverse events, there is a decrease in risk among younger adults compared to older adults. The 65–74 and 75–84 age groups both show a reduced risk (0.89 [0.84–0.94], 0.89 [0.83–0.95]), with the 85+ age group having an even lower risk (0.79 [0.66–0.93]) relative to the 18–64 age group. In contrast, renal-related adverse events exhibit an increased risk in older adults. The 65–74, 75–84 and 85+ age groups show elevated risks compared to the 18–64 age group (Figure 2). Finally, our analysis revealed no significant changes in toxicity for immune-related cutaneous, neurological and haematological adverse events across all age groups.

Table 2 presents adjusted ORs from a multivariate logistic regression analysing the association of ICI treatment regimens with adverse effects (AEs) across age groups. The risk of irAEs with ICI monotherapy significantly increases with age, particularly in the 65–74 and 75–84 age groups (1.13 [1.09–1.18], 1.15 [1.1–1.21]), compared to younger adults. However, there are no significant changes in risk between the 18–64 age group and the 85+ age group, or among older age comparisons such as 65–74 versus older groups and 75–84 versus 85+ (Table 2).

**Table 2:** The ORs and *P*-values for the multivariate regression for the five most commonly used treatment regimens comparing the patients with irAEs in age group pairs

Treatment regimen	Age 18–64 vs. 65–74	Age 18–64 vs. 75–84	Age 18–64 vs. 85+	Age 65–74 vs. 75–84	Age 65–74 vs. 85+	Age 75–84 vs. 85+
**ICI monotherapy**	**1.13 [1.09–1.18]**	**1.15 [1.1–1.21]**	1.07 [0.97–1.19]	1.01 [0.96–1.06]	0.93 [0.84–1.03]	0.92 [0.83–1.02]
Anti-PD1	**1.18 [1.13–1.23]**	**1.19 [1.13–1.25]**	**1.12 [1–1.25]**	0.99 [0.94–1.05]	0.92 [0.82–1.03]	0.92 [0.82–1.03]
Anti-PD-L1	1.03 [0.94–1.12]	1.09 [0.98–1.21]	0.96 [0.74–1.25]	1.09 [0.98–1.21]	0.95 [0.73–1.23]	0.87 [0.66–1.14]
Anti-CTLA4	0.98 [0.81–1.17]	1.11 [0.88–1.41]	1.35 [0.71–2.71]	1.18 [0.92–1.53]	1.43 [0.74–2.90]	1.25 [0.64–2.57]
**ICI dual therapy**	1.03 [0.96–1.11]	1.06 [0.96–1.17]	**0.65 [0.49–0.85]**	1.03 [0.93–1.14]	**0.62 [0.47–0.82]**	**0.61 [0.46–0.81]**

Data are presented in ‘OR, [CI]’ format. The significant results are in bold. ICI, immune checkpoint inhibitor; NA, not applicable.

The same pattern is observed specifically within the anti-PD-1 monotherapy subgroup, where older age groups exhibit a higher risk of irAEs when compared to the 18–64 age group, mirroring the overall ICI monotherapy findings but with an additional notable increase in risk in the 85+ age group compared to the 18–64 age group. However, there is no significant change when comparing between the older adult age groups themselves. For the anti-PD-L1 and anti-CTLA-4 monotherapy subgroups, our analysis shows no significant risk changes across all age comparisons.

In the dual therapy subgroup, a significant decrease in the risk of irAEs was observed in the 85+ age group compared to younger cohorts, with ORs of 0.65 [0.49–0.85] for 18–64 versus 85+, 0.62 [0.47–0.82] for 65–74 versus 85+, and 0.61 [0.46–0.81] for 75–84 versus 85+. However, comparisons within younger age groups, such as 18–64 versus 65–74, 18–64 versus 75–84 and 65–74 versus 75–84, showed no significant change in the risk of irAEs (Table 2).

## Discussion

In our analysis of the FAERS dataset, we observed that patients younger than 65 years of age and those aged 85 or older experienced fewer irAEs compared to the 65–75 and 75–85 age groups. This suggests that irAE incidence does not uniformly change with age, challenging simple age-irAE risk correlations. Literature on this topic is mixed, with several studies using a single age cut-off to explore this relationship, often finding that older patients may have similar or lower irAE incidences [19, 30–34]. However, these studies are mainly observational with small sample sizes, limiting generalisability. Two retrospective analyses have found no significant difference in irAE rates among their respective age groups (<65, 65–75, >75 and < 85, 85–89, >90) [35, 36]. Conversely, a similar study that utilised the FAERS database with a single cut-off at 65 years reported an age-related increase in irAE incidence. [29] Our findings partially corroborate this, indicating higher irAE rates in the 65–74 and 75–84 groups but lower rates in those over 85.

The variation in irAE prevalence across different age groups may be influenced by immunosenescence, which involves a decline in immune function, alterations in T-cell subsets and dysregulation of immune homeostasis. These alterations can affect the interaction between the immune system and immune-modulating drugs like ICIs, potentially impacting their efficacy and toxicity in older patients [37]. However, as the FAERS database lacks immunophenotypic data, our study cannot directly assess patients’ immunological profiles or markers of immune senescence. Since examining these processes in a large-scale analysis remains challenging, we utilise age only as a surrogate marker for immunosenescence and physiological ageing within a dataset of this size.

The pattern of fewer irAEs in the 85+ age group could be attributed to survivorship bias. Patients in this age range eligible for ICI therapy may represent a particularly resilient subset with fewer comorbidities, resulting in a higher tolerance to ICI AEs. Additionally, oncologists may opt for more conservative treatment plans or lower ICI dosages for the oldest patients, considering their overall frailty, which could naturally reduce the incidence of adverse events. The observed decrease in dual therapy, additional targeted therapies and chemotherapy usage with increasing age further underscore the potential impact of selective therapy. Underreporting is another factor that could contribute to this finding, especially in the oldest age group. Differentiating between age-related symptoms or comorbidities and genuine ICI-related side effects can be challenging, potentially leading to the misattribution of irAEs as normal age-related conditions. Unfortunately, the limitations of FAERS data prevent comprehensive consideration of these factors in our analysis. However, to strengthen our results, we meticulously selected PTs focused on well-characterised adverse events with established diagnostic and treatment guidelines, aiming to minimise the risk of age-related underrecognition of side effects. Furthermore, the finding of a lower incidence of side effects in the 85–100 age group among dual therapy users indicates a true reduction rather than solely the impact of a selection bias.

Our study revealed age-specific trends in the incidence of irAEs across different organ systems. We noted a decrease in irAEs related to the endocrine, eye, gastrointestinal and hepatobiliary systems with age, while irAEs in the musculoskeletal, renal and cardiovascular systems increased. These variations likely result from distinct mechanisms, such as autoantibody activity in thyroid irAEs, T-cell mediation in myocarditis and cytokine involvement in colitis [38].

Corroborating our findings, one study found immune-related hepatitis more common in patients under 55 during dual ICI therapy, while another observed a higher incidence of acute kidney injury with ICIs in older individuals [39, 40]. Additionally, one report noted a 23% incidence of endocrine irAEs in patients treated with ICIs, identifying being under 61 as a significant risk factor; another found a lower incidence in those over 75 [35, 41]. The infrequent occurrence of endocrine irAEs in older individuals might be due to hormonal declines and the role of endocrine-specific autoantibodies [42, 43]. Conversely, the rise in cardiovascular irAEs among older patients could stem from age-related physiological changes and T-cell senescence affecting cardiovascular health [44].

Further research has shed light on irAE severity and regimen discontinuation patterns. One study showed that despite similar irAE rates, patients aged 90 and older had a higher frequency of irAE-induced ICI discontinuation compared to those 80–89, even for lower-grade irAEs [36]. Another study noted a rise in severe complications, including fatal irAEs, in those 75 and older [31]. Meanwhile, our findings indicated a modest, nonsignificant decrease in serious outcomes but an increase in the proportion of deaths with age. These results highlight the importance of considering additional outcomes like ICI discontinuation and irAE severity in clinical decision-making.

As evident from our results, age alone should not encourage or discourage the use of ICIs. Geriatric assessment tools like the Geriatric 8 (G8) and Eastern Cooperative Oncology Group (ECOG) Performance Status are being investigated for predicting ICI therapy outcomes in older patients [45, 46]. The G8, a multidimensional tool for older cancer patients, has revealed that frail patients are more likely to be hospitalised for irAEs and discontinue ICIs, while ECOG status significantly influences outcomes like treatment discontinuation and hospitalisation, rather than age [47, 48]. The ELDERS study also confirmed that a positive G8 screening correlates with higher hospital admissions and mortality rates in ICI users [49]. These insights emphasise the importance of comprehensive geriatric assessments and personalised treatment strategies based on more than chronological age for older patients on ICIs.

Our study had several strengths and limitations. It benefits from a large sample size and is, to our knowledge, the first to analyse the FAERS database for irAEs with ICI use in older patients, including detailed subgroup analyses. However, limitations included a lack of data on dosage variations, drug discontinuation and therapy duration. Additionally, as the FAERS database lacks information on immunophenotypic markers, we were unable to explore the mechanistic aspects underlying our results. The potential underreporting of adverse events in older populations and the rigorous selection process possibly applied to these patients were not fully addressed in our analysis.

To conclude, our study highlights the intricate relationship between age, immune function and irAE incidence in patients on ICI therapy, using real-world data from the FAERS database. Our findings suggest that age alone should not preclude the use of ICIs in older patients. Drawing from the literature, we recommend incorporating geriatric assessments into clinical decision-making to customise immunotherapy strategies. Further research is needed to explore dosage variations and the unique challenges of treating the oldest patient groups.

## Supplementary Material

aa-24-1110-File002_afaf008
