# Supplementary material for: Immune-related adverse events in older adults receiving immune checkpoint inhibitors: a comprehensive analysis of the Food and Drug Administration Adverse Event Reporting System
Source: Age Ageing. 2025 Jan 30;54(1):afaf008. doi: 10.1093/ageing/afaf008 (PMC11781319; doi:10.1093/ageing/afaf008)
Supplement: aa-24-1110-File002_afaf008 [file aa-24-1110-file002_afaf008.docx]

**Supplementary Appendix**

**Immune-related adverse events in older adults receiving immune checkpoint inhibitors: a comprehensive analysis of the Food and Drug Administration (FDA) Adverse Event Reporting System**

**Table of Contents**

- **Supplementary Table S1**. List of included preferred terms (PT) below the standardized MedDRA queries (SMQs) of reproductive adverse reactions
- **Supplementary Table S2**. List of chemotherapies with the international nonproprietary names (INNs)
- **Supplementary Table S3**. List of targeted therapies with the international nonproprietary names (INNs)
- **Supplementary Table S4.** List of reported countries, their income levels and number of reported non-irAE and irAE cases
- **Supplementary Figure F1.** The rates of overall and system associated irAEs in 18-64, 65-74, 75-84 and 85-100 age groups.
- **Supplementary Table S5.** Univariate Analysis of Factors Influencing Immune-Related Adverse Events

# Supplementary Table S1. List of included preferred terms (PT) below the standardized MedDRA queries (SMQs) of reproductive adverse reactions

| **Hematological irAEs** | Thyroid atrophy | Cholecystitis | Small fibre neuropathy |
| --- | --- | --- | --- |
| Autoimmune haemolytic anaemia | Blood prolactin decreased | Transaminases increased | Optic neuropathy |
| Evans syndrome | **Ophthalmological irAEs** | Cholecystitis acute | Trigeminal palsy |
| Warm autoimmune haemolytic anaemia | Noninfective conjunctivitis | Liver function test increased | Trigeminal neuritis |
| Cold type haemolytic anaemia | Keratitis | Haemorrhagic cholecystitis | Neurosarcoidosis |
| Haemophagocytic lymphohistiocytosis | Ulcerative keratitis | Liver function test abnormal | **Renal irAEs** |
| Coombs positive haemolytic anaemia | Punctate keratitis | Hepatic enzyme abnormal | Blood creatinine increased |
| Haemolytic uraemic syndrome | Uveitis | Aspartate aminotransferase abnormal | Blood creatinine abnormal |
| Microangiopathic haemolytic anaemia | Immune-mediated uveitis | Hepatic function abnormal | Nephrotic syndrome |
| Atypical haemolytic uraemic syndrome | Iridocyclitis | Hypertransaminasaemia | IgA nephropathy |
| Haemolytic anaemia | Vogt-Koyanagi-Harada disease | Hepatic failure | Glomerulonephritis membranous |
| Coombs negative haemolytic anaemia | Iritis | Acute hepatic failure | Glomerulonephritis |
| Autoimmune anaemia | Autoimmune uveitis | Subacute hepatic failure | Glomerulonephritis rapidly progressive |
| Acquired haemophilia | Dry eye | Primary biliary cholangitis | Glomerulonephritis minimal lesion |
| Acquired factor VIII deficiency | Blepharitis | Transaminases abnormal | Nephritis allergic |
| Antiphospholipid syndrome | Conjunctivitis | Hepatitis | Glomerulonephritis proliferative |
| Leukopenia | Autoimmune retinopathy | Immune-mediated hepatitis | Glomerulonephritis membranoproliferative |
| Lymphopenia | Scleritis | Drug-induced liver injury | Goodpasture's syndrome |
| Basophilopenia | Episcleritis | Immune-mediated hepatic disorder | Focal segmental glomerulosclerosis |
| Myelosuppression | Immune-mediated scleritis | Autoimmune hepatitis | Henoch-Schonlein purpura nephritis |
| Pancytopenia | Ocular sarcoidosis | Hepatotoxicity | Glomerulonephritis acute |
| Bone marrow failure | Autoimmune eye disorder | Hepatocellular injury | Anti-glomerular basement membrane disease |
| Aplasia pure red cell | Chorioretinitis | Hepatic cytolysis | Glomerulonephritis chronic |
| Cytopenia | Retinitis | Liver injury | Mesangioproliferative glomerulonephritis |
| Aplastic anaemia | Keratouveitis | Hepatitis acute | IgM nephropathy |
| Febrile bone marrow aplasia | **Gastrointestinal irAEs** | Hepatitis fulminant | C3 glomerulopathy |
| Bicytopenia | Pancreatitis | Hepatitis toxic | Tubulointerstitial nephritis |
| Immune-mediated cytopenia | Pancreatitis acute | **Musculoskeletal irAEs** | Nephritis |
| Autoimmune pancytopenia | Immune-mediated pancreatitis | Arthritis | Immune-mediated nephritis |
| Autoimmune aplastic anaemia | Autoimmune pancreatitis | Polyarthritis | Autoimmune nephritis |
| Pure white cell aplasia | Pancreatitis chronic | Immune-mediated arthritis | Lupus nephritis |
| Immune-mediated pancytopenia | Pancreatitis necrotising | Autoimmune arthritis | Immune-mediated renal disorder |
| Febrile neutropenia | Oedematous pancreatitis | Seronegative arthritis | Glomerulonephropathy |
| Neutropenia | Pancreatitis haemorrhagic | Oligoarthritis | Glomerulosclerosis |
| Agranulocytosis | Pancreatic phlegmon | Sacroiliitis | Acute kidney injury |
| Autoimmune neutropenia | Lipase increased | Sarcoidosis | Renal failure |
| Granulocytopenia | Amylase increased | Dermatomyositis | Renal impairment |
| Thrombocytopenia | Rectal ulcer | Paraneoplastic dermatomyositis | **Pulmonary irAEs** |
| Immune thrombocytopenia | Rectal perforation | Giant cell arteritis | Pneumonitis |
| Thrombotic thrombocytopenic purpura | Anal ulcer | Arteritis | Immune-mediated lung disease |
| Thrombocytopenic purpura | Rectal ulcer haemorrhage | Takayasu's arteritis | Hypersensitivity pneumonitis |
| Acquired amegakaryocytic thrombocytopenia | Colitis | Sjogren's syndrome | Pulmonary sarcoidosis |
| Acquired factor V deficiency | Immune-mediated enterocolitis | Polymyalgia rheumatica | Eosinophilic pneumonia |
| Acquired Von Willebrand's disease | Autoimmune colitis | Eosinophilic fasciitis | Alveolitis |
| **Cardiovascular irAEs** | Colitis ulcerative | Central nervous system vasculitis | Diffuse alveolar damage |
| Atrioventricular block complete | Colitis microscopic | Scleroderma | Autoimmune lung disease |
| Atrioventricular block | Enterocolitis haemorrhagic | Morphoea | Eosinophilic pneumonia acute |
| Bundle branch block right | Crohn's disease | Systemic scleroderma | Diffuse panbronchiolitis |
| Atrioventricular block second degree | Inflammatory bowel disease | Scleroderma-like reaction | Interstitial lung disease |
| Bundle branch block left | Neutropenic colitis | Immunoglobulin G4 related disease | Organising pneumonia |
| Atrioventricular block first degree | Allergic colitis | Myofascitis | Idiopathic pulmonary fibrosis |
| Bundle branch block | Colitis erosive | Mixed connective tissue disease | Acute interstitial pneumonitis |
| Atrioventricular dissociation | Terminal ileitis | Pulmonary vasculitis | Idiopathic interstitial pneumonia |
| Ejection fraction decreased | Eosinophilic colitis | Anti-neutrophil cytoplasmic antibody positive vasculitis | Pulmonary granuloma |
| Cardiac failure | Acute haemorrhagic ulcerative colitis | Eosinophilic granulomatosis with polyangiitis | Granulomatous pneumonitis |
| Cardiac failure congestive | Necrotising colitis | Systemic lupus erythematosus | **Cutaneous irAEs** |
| Cardiogenic shock | Pancreatic enzymes increased | Lupus-like syndrome | Dermatitis acneiform |
| Cardiac failure acute | Diarrhoea | Vasculitis gastrointestinal | Alopecia |
| Cardiac failure chronic | Diarrhoea haemorrhagic | Myositis | Alopecia areata |
| Low cardiac output syndrome | Duodenal ulcer | Immune-mediated myositis | Alopecia universalis |
| Ventricular failure | Duodenal perforation | Polymyositis | Pemphigoid |
| Left ventricular failure | Duodenal ulcer haemorrhage | Autoimmune myositis | Stevens-Johnson syndrome |
| Acute left ventricular failure | Gastroduodenal ulcer | Antisynthetase syndrome | Erythema multiforme |
| Left ventricular dysfunction | Duodenal ulcer perforation | Inclusion body myositis | Blister |
| Myocardial injury | Ulcerative duodenitis | Myositis-like syndrome | Toxic epidermal necrolysis |
| Ventricular hypokinesia | Erosive duodenitis | Idiopathic inflammatory myopathy | Dermatitis bullous |
| Ventricular dysfunction | Gastric haemorrhage | Focal myositis | Pemphigus |
| Myocardial fibrosis | Gastroduodenal haemorrhage | Orbital myositis | Epidermolysis |
| Myocardial necrosis | Gastric ulcer | Henoch-Schonlein purpura | Autoimmune blistering disease |
| Systolic dysfunction | Gastric perforation | Renal vasculitis | Acquired epidermolysis bullosa |
| Cardiac sarcoidosis | Gastric ulcer haemorrhage | Necrotising myositis | SJS-TEN overlap |
| Myocarditis | Gastritis erosive | Psoriatic arthropathy | Mucous membrane pemphigoid |
| Immune-mediated myocarditis | Gastric ulcer perforation | Rheumatoid arthritis | Paraneoplastic pemphigus |
| Autoimmune myocarditis | Ulcerative gastritis | Still's disease | Blood blister |
| Myopericarditis | Haemorrhagic erosive gastritis | Retinal vasculitis | Dermatitis herpetiformis |
| Eosinophilic myocarditis | Gastritis | Ankylosing spondylitis | Lichen planus pemphigoides |
| Giant cell myocarditis | Chronic gastritis | Spondylitis | Linear IgA disease |
| Pericarditis | Immune-mediated gastritis | Arthritis reactive | Generalised bullous fixed drug eruption |
| Autoimmune pericarditis | Gastritis haemorrhagic | Spondyloarthropathy | Subacute cutaneous lupus erythematosus |
| Immune-mediated pericarditis | Eosinophilic gastritis | Axial spondyloarthritis | Cutaneous lupus erythematosus |
| Pericarditis constrictive | Necrotising gastritis | Synovitis | Chronic cutaneous lupus erythematosus |
| Pericardial effusion | Enterocolitis | Vasculitis | Skin discolouration |
| Cardiac tamponade | Enteritis | Granulomatosis with polyangiitis | Neutrophilic dermatosis |
| Pericardial haemorrhage | Duodenitis | Behcet's syndrome | Dermatitis |
| Pericardial fibrosis | Gastroenteritis eosinophilic | Cutaneous vasculitis | Immune-mediated dermatitis |
| Arrhythmia | Mesenteric panniculitis | Hypersensitivity vasculitis | Eczema |
| Tachyarrhythmia | Autoimmune enteropathy | Tenosynovitis | Dermatitis allergic |
| Cardiac flutter | Duodenitis haemorrhagic | Vasculitis necrotising | Autoimmune dermatitis |
| Bradyarrhythmia | Microscopic enteritis | Polyarteritis nodosa | Eczema asteatotic |
| Cardiac fibrillation | Lipase abnormal | Diffuse vasculitis | Prurigo |
| Atrial fibrillation | Gastrointestinal necrosis | Microscopic polyangiitis | Neurodermatitis |
| Supraventricular tachycardia | Gastrointestinal mucosal necrosis | Connective tissue disorder | Superficial inflammatory dermatosis |
| Atrial flutter | Gastrointestinal perforation | Cryoglobulinaemia | Hand dermatitis |
| Sinus bradycardia | Gastrointestinal ulcer | Susac's syndrome | Perioral dermatitis |
| Sinus node dysfunction | Gastrointestinal erosion | Rheumatoid vasculitis | Eczema nummular |
| Arrhythmia supraventricular | Gastrointestinal ulcer haemorrhage | **Neurological irAEs** | Dermatitis atopic |
| Atrial tachycardia | Upper gastrointestinal perforation | Guillain-Barre syndrome | Dyshidrotic eczema |
| Ventricular tachycardia | Gastrointestinal ulcer perforation | Acute polyneuropathy | Eczema vesicular |
| Ventricular fibrillation | Rectal haemorrhage | Miller Fisher syndrome | Drug eruption |
| Ventricular arrhythmia | Lower gastrointestinal haemorrhage | Acute motor axonal neuropathy | Palmar-plantar erythrodysaesthesia syndrome |
| Ventricular tachyarrhythmia | Small intestinal haemorrhage | Acute motor-sensory axonal neuropathy | Toxic skin eruption |
| Accelerated idioventricular rhythm | Intestinal haemorrhage | Subacute inflammatory demyelinating polyneuropathy | Drug reaction with eosinophilia and systemic symptoms |
| Sinus arrhythmia | Large intestinal haemorrhage | Autonomic neuropathy | Fixed eruption |
| Nodal arrhythmia | Large intestine perforation | Demyelinating polyneuropathy | Symmetrical drug-related intertriginous and flexural exanthema |
| **Endocrine irAEs** | Intestinal perforation | Chronic inflammatory demyelinating polyradiculoneuropathy | Skin exfoliation |
| Thyroiditis | Small intestinal perforation | Polyneuropathy in malignant disease | Dermatitis exfoliative generalised |
| Autoimmune thyroiditis | Large intestinal ulcer | Cranial nerve paralysis | Dermatitis exfoliative |
| Immune-mediated thyroiditis | Jejunal perforation | Cranial nerve palsies multiple | Exfoliative rash |
| Silent thyroiditis | Ileal perforation | Neuritis cranial | Cutaneous sarcoidosis |
| Thyroiditis subacute | Jejunal ulcer | Demyelination | Granuloma annulare |
| Thyroiditis chronic | Lower gastrointestinal perforation | Neuromyelitis optica spectrum disorder | Granulomatous dermatitis |
| Thyroiditis acute | Small intestine ulcer | Acute disseminated encephalomyelitis | Interstitial granulomatous dermatitis |
| Blood thyroid stimulating hormone increased | Intestinal ulcer | Encephalitis | Granuloma skin |
| Adrenal insufficiency | Large intestinal ulcer perforation | Autoimmune demyelinating disease | Lichenoid keratosis |
| Immune-mediated adrenal insufficiency | Large intestinal ulcer haemorrhage | Myelin oligodendrocyte glycoprotein antibody-associated disease | Hyperkeratosis |
| Type 1 diabetes mellitus | Large intestine erosion | Meningitis aseptic | Palmoplantar keratoderma |
| Cortisol decreased | Ileal ulcer | Myelitis | Keratosis pilaris |
| Blood thyroid stimulating hormone decreased | Ileal ulcer perforation | Encephalomyelitis | Vitiligo |
| Adrenocortical insufficiency acute | Small intestinal ulcer perforation | Encephalitis autoimmune | Leukoderma |
| Fulminant type 1 diabetes mellitus | Amylase abnormal | Immune-mediated encephalitis | Skin depigmentation |
| Addison's disease | Gastrointestinal haemorrhage | Noninfective encephalitis | Skin hypopigmentation |
| Glucocorticoid deficiency | Haematochezia | Paraneoplastic encephalomyelitis | Lichen planus |
| Thyroid function test abnormal | Melaena | Bickerstaff's encephalitis | Lichen sclerosus |
| Primary adrenal insufficiency | Upper gastrointestinal haemorrhage | Immune-mediated encephalopathy | Lichenification |
| Latent autoimmune diabetes in adults | Haematemesis | Autoimmune encephalopathy | Pityriasis rubra pilaris |
| Blood corticotrophin decreased | Oesophageal ulcer | VIth nerve paralysis | Acrokeratosis paraneoplastica |
| Diabetic ketoacidosis | Oesophageal perforation | IIIrd nerve paralysis | Parapsoriasis |
| Diabetic ketosis | Necrotising oesophagitis | IIIrd nerve paresis | Erythema annulare |
| Blood thyroid stimulating hormone abnormal | Oesophagitis ulcerative | VIth nerve disorder | Pigmentation disorder |
| Thyroid hormones increased | Oesophageal rupture | IIIrd nerve disorder | Pruritus |
| Thyroxine free decreased | Erosive oesophagitis | IVth nerve paralysis | Psoriasis |
| Hypopituitarism | Oesophagitis | VIth nerve paresis | Dermatitis psoriasiform |
| Adrenocorticotropic hormone deficiency | Eosinophilic oesophagitis | IVth nerve paresis | Pustular psoriasis |
| Diabetic ketoacidotic hyperglycaemic coma | Immune-mediated oesophagitis | Facial paralysis | Guttate psoriasis |
| Thyroxine free increased | Lymphocytic oesophagitis | Facial paresis | Erythrodermic psoriasis |
| Thyroid hormones decreased | Pancreatic failure | Bell's palsy | Palmoplantar pustulosis |
| Thyroid stimulating hormone deficiency | Proctitis | Facial nerve disorder | Acute generalised exanthematous pustulosis |
| Growth hormone deficiency | Proctitis ulcerative | Hypoglossal nerve paralysis | Rash follicular |
| Thyroxine increased | Proctitis haemorrhagic | Pachymeningitis | Rash |
| Apituitarism | Stomatitis | Meningitis noninfective | Rash maculo-papular |
| Thyroxine free abnormal | Mouth ulceration | Bulbar palsy | Rash pruritic |
| Thyroxine decreased | Aphthous ulcer | Myelitis transverse | Rash erythematous |
| Hypophysitis | Oral mucosa erosion | Paraneoplastic myelopathy | Rash macular |
| Secondary adrenocortical insufficiency | Lip erosion | Immune-mediated myelitis | Rash papular |
| Lymphocytic hypophysitis | Lip ulceration | Noninfectious myelitis | Rash vesicular |
| Immune-mediated hypophysitis | Stomatitis necrotising | Paraneoplastic neurological syndrome | Rash morbilliform |
| Blood luteinising hormone decreased | Stomatitis haemorrhagic | Ocular myasthenia | Butterfly rash |
| Blood follicle stimulating hormone decreased | Gastrointestinal tract irritation | Myasthenia gravis | Heliotrope rash |
| Secondary hypogonadism | Gastrointestinal mucosa hyperaemia | Myasthenic syndrome | Mucocutaneous rash |
| Thyroxine abnormal | Cryptitis | Immune-mediated myasthenia gravis | Skin ulcer |
| Diabetes insipidus | Frequent bowel movements | Myasthenia gravis crisis | Skin erosion |
| Autoimmune thyroid disorder | Palatal ulcer | Optic neuritis | Pyoderma gangrenosum |
| Hyperthyroidism | **Hepatobiliary irAEs** | Optic perineuritis | Skin ulcer haemorrhage |
| Immune-mediated hyperthyroidism | Alanine aminotransferase abnormal | Immune-mediated optic neuritis | Acute febrile neutrophilic dermatosis |
| Graves' disease | Cholangitis | Neuropathy peripheral | Acute cutaneous lupus erythematosus |
| Thyrotoxic crisis | Alanine aminotransferase increased | Peripheral sensory neuropathy | Severe cutaneous adverse reaction |
| Primary hyperthyroidism | Aspartate aminotransferase increased | Polyneuropathy | Sarcoid-like reaction |
| Toxic nodular goitre | Cholangitis sclerosing | Peripheral motor neuropathy | Panniculitis |
| Hypothyroidism | Immune-mediated cholangitis | Immune-mediated neuropathy | Erythema nodosum |
| Immune-mediated hypothyroidism | Cholangitis acute | Neuritis | Panniculitis lobular |
| Autoimmune hypothyroidism | Autoimmune cholangitis | Autoimmune neuropathy | Septal panniculitis |
| Central hypothyroidism | Cholangiolitis | Peripheral sensorimotor neuropathy | Neutrophilic panniculitis |
| Primary hypothyroidism | Cholecystocholangitis | Axonal neuropathy | Rash papulosquamous |
| Myxoedema | Hepatic enzyme increased | Axonal and demyelinating polyneuropathy |  |

# Supplementary Table S2. List of chemotherapies with the international nonproprietary names (INNs)

Amrubicin, Asparaginase, Azacitidine, Bendamustine, Bleomycin, Busulfan, Cabazitaxel, Capecitabine, Carboplatin, Carmustine, Chlorambucil, Cisplatin, Cyclophosphamide, Cytarabine, Dacarbazine, Dactinomycin, Decitabine, Docetaxel, Doxorubicin, Epirubicin, Eribulin, Etoposide, Fluorouracil, Fotemustine, Gemcitabine, Idarubicin, Ifosfamide, Irinotecan, Lomustine, Lurbinectedin, Melphalan, Mercaptopurine, Methotrexate, Mitomycin, Mitotane, Nelarabine, Nimustine, Oxaliplatin, Paclitaxel, Pemetrexed, Pirarubicin, Procarbazine, Raltitrexed, Ranimustine, Tegafur, Temozolomide, Thiotepa, Topotecan, Trabectedin, Trofosfamide, Vinblastine, Vincristine, Vinflunine, Vinorelbine

# Supplementary Table S3. List of targeted therapies with the international nonproprietary names (INNs)

Abemaciclib, Acalabrutinib, Adagrasib, Afatinib, Aflibercept, Alectinib, Alpelisib, Arsenic trioxide, Aumolertinib, Avapritinib, Axitinib, Belantamab mafodotin, Belinostat, Belzutifan, Bevacizumab, Binimetinib, Blinatumomab, Bortezomib, Bosutinib, Brentuximab Vedotin, Brigatinib, Cabozantinib, Capmatinib, Carfilzomib, Cediranib, Celecoxib, Ceritinib, Cetuximab, Cobimetinib, Copanlisib, Crizotinib, Dabrafenib, Daratumumab, Dasatinib, Dinutuximab beta, Duvelisib, Elotuzumab, Encorafenib, Enfortumab vedotin, Entinostat, Entrectinib, Erdafitinib, Erlotinib, Everolimus, Gefitinib, Gilteritinib, Ibrutinib, Idelalisib, Imatinib, Isatuximab, Ivosidenib, Ixazomib, Lapatinib, Lenvatinib, Lorlatinib, Mobocertinib, Mogamulizumab, Necitumumab, Neratinib, Nilotinib, Nintedanib, Niraparib, Obinutuzumab, Olaparib, Olaratumab, Osimertinib, Palbociclib, Pamiparib, Panitumumab, Panobinostat, Pazopanib, Pemigatinib, Pertuzumab, Pexidartinib, Polatuzumab vedotin, Ponatinib, Pralsetinib, Ramucirumab, Regorafenib, Ribociclib, Ripretinib, Rituximab, Romidepsin, Rucaparib, Ruxolitinib, Sacituzumab, Sacituzumab govitecan, Selinexor, Selumetinib, Sirolimus, Sorafenib, Sotorasib, Sunitinib, Surufatinib, Talazoparib, Tazemetostat, Tebentafusp, Temsirolimus, Tepotinib, Tisotumab vedotin, Tivozanib, Trametinib, Trastuzumab, Trastuzumab deruxtecan, Tucatinib, Vandetanib, Veliparib, Vemurafenib, Venetoclax, Vismodegib, Vorinostat, Zanubrutinib

# Supplementary Table S4. List of reported countries, their income levels and number of reported non-irAE and irAE cases

| **Country** | **Income Level** | **Total number of reports** | **Patients aged 18-64** | | **Patients aged 65-74** | | **Patients aged 75-84** | | **Patients aged 85-100** | |
| --- | --- | --- | --- | --- | --- | --- | --- | --- | --- | --- |
|  |  |  | **Non-irAE cases** | **irAE cases** | **Non-irAE cases** | **irAE cases** | **Non-irAE cases** | **irAE cases** | **Non-irAE cases** | **irAE cases** |
| United States | High Income | 24753 | 6780 (27.4%) | 5099 (20.6%) | 4156 (16.8%) | 3682 (14.9%) | 2316 (9.4%) | 1844 (7.4%) | 550 (2.2%) | 326 (1.3%) |
| Japan | High Income | 20628 | 1987 (9.6%) | 4248 (20.6%) | 2935 (14.2%) | 5702 (27.6%) | 1800 (8.7%) | 3403 (16.5%) | 193 (0.9%) | 360 (1.7%) |
| France | High Income | 8260 | 1561 (18.9%) | 2086 (25.3%) | 1251 (15.1%) | 1609 (19.5%) | 635 (7.7%) | 865 (10.5%) | 95 (1.2%) | 158 (1.9%) |
| Germany | High Income | 4194 | 839 (20.0%) | 1109 (26.4%) | 535 (12.8%) | 793 (18.9%) | 383 (9.1%) | 465 (11.1%) | 34 (0.8%) | 36 (0.9%) |
| China | Upper Middle Income | 2971 | 949 (31.9%) | 736 (24.8%) | 575 (19.4%) | 419 (14.1%) | 160 (5.4%) | 111 (3.7%) | 18 (0.6%) | 3 (0.1%) |
| Italy | High Income | 2350 | 413 (17.6%) | 534 (22.7%) | 349 (14.9%) | 539 (22.9%) | 183 (7.8%) | 299 (12.7%) | 12 (0.5%) | 21 (0.9%) |
| Canada | High Income | 2086 | 508 (24.4%) | 545 (26.1%) | 348 (16.7%) | 335 (16.1%) | 142 (6.8%) | 171 (8.2%) | 23 (1.1%) | 14 (0.7%) |
| Australia | High Income | 1812 | 390 (21.5%) | 417 (23.0%) | 313 (17.3%) | 279 (15.4%) | 189 (10.4%) | 163 (9.0%) | 33 (1.8%) | 28 (1.5%) |
| United Kingdom | High Income | 1776 | 389 (21.9%) | 469 (26.4%) | 262 (14.8%) | 332 (18.7%) | 111 (6.2%) | 186 (10.5%) | 10 (0.6%) | 17 (1.0%) |
| Spain | High Income | 1610 | 319 (19.8%) | 450 (28.0%) | 199 (12.4%) | 352 (21.9%) | 102 (6.3%) | 162 (10.1%) | 10 (0.6%) | 16 (1.0%) |
| India | Lower Middle Income | 940 | 566 (60.2%) | 55 (5.9%) | 202 (21.5%) | 31 (3.3%) | 67 (7.1%) | 11 (1.2%) | 8 (0.9%) | 0 (0.0%) |
| Belgium | High Income | 776 | 190 (24.5%) | 200 (25.8%) | 105 (13.5%) | 154 (19.8%) | 54 (7.0%) | 65 (8.4%) | 3 (0.4%) | 5 (0.6%) |
| Korea | High Income | 720 | 236 (32.8%) | 171 (23.8%) | 142 (19.7%) | 95 (13.2%) | 41 (5.7%) | 28 (3.9%) | 5 (0.7%) | 2 (0.3%) |
| Netherlands | High Income | 710 | 191 (26.9%) | 167 (23.5%) | 126 (17.7%) | 113 (15.9%) | 51 (7.2%) | 51 (7.2%) | 4 (0.6%) | 7 (1.0%) |
| Switzerland | High Income | 643 | 100 (15.6%) | 194 (30.2%) | 86 (13.4%) | 139 (21.6%) | 44 (6.8%) | 66 (10.3%) | 5 (0.8%) | 9 (1.4%) |
| Brazil | Upper Middle Income | 623 | 175 (28.1%) | 170 (27.3%) | 81 (13.0%) | 93 (14.9%) | 40 (6.4%) | 44 (7.1%) | 11 (1.8%) | 9 (1.4%) |
| Taiwan | High Income | 563 | 146 (25.9%) | 153 (27.2%) | 102 (18.1%) | 79 (14.0%) | 32 (5.7%) | 39 (6.9%) | 6 (1.1%) | 6 (1.1%) |
| Israel | High Income | 520 | 105 (20.2%) | 116 (22.3%) | 96 (18.5%) | 92 (17.7%) | 46 (8.8%) | 45 (8.7%) | 8 (1.5%) | 12 (2.3%) |
| Poland | High Income | 480 | 131 (27.3%) | 119 (24.8%) | 90 (18.8%) | 83 (17.3%) | 20 (4.2%) | 31 (6.5%) | 2 (0.4%) | 4 (0.8%) |
| Austria | High Income | 459 | 87 (19.0%) | 108 (23.5%) | 65 (14.2%) | 83 (18.1%) | 45 (9.8%) | 59 (12.9%) | 4 (0.9%) | 8 (1.7%) |
| Greece | High Income | 367 | 70 (19.1%) | 80 (21.8%) | 52 (14.2%) | 84 (22.9%) | 40 (10.9%) | 32 (8.7%) | 7 (1.9%) | 2 (0.5%) |
| Czechia | High Income | 360 | 56 (15.6%) | 92 (25.6%) | 53 (14.7%) | 114 (31.7%) | 16 (4.4%) | 28 (7.8%) | 0 (0.0%) | 1 (0.3%) |
| Russia | Upper Middle Income | 360 | 145 (40.3%) | 88 (24.4%) | 64 (17.8%) | 41 (11.4%) | 9 (2.5%) | 12 (3.3%) | 0 (0.0%) | 1 (0.3%) |
| Turkey | Upper Middle Income | 332 | 141 (42.5%) | 80 (24.1%) | 62 (18.7%) | 30 (9.0%) | 13 (3.9%) | 5 (1.5%) | 1 (0.3%) | 0 (0.0%) |
| Ireland | High Income | 328 | 83 (25.3%) | 88 (26.8%) | 52 (15.9%) | 48 (14.6%) | 28 (8.5%) | 25 (7.6%) | 1 (0.3%) | 3 (0.9%) |
| Colombia | Upper Middle Income | 317 | 121 (38.2%) | 43 (13.6%) | 65 (20.5%) | 22 (6.9%) | 45 (14.2%) | 14 (4.4%) | 5 (1.6%) | 2 (0.6%) |
| Mexico | Upper Middle Income | 303 | 106 (35.0%) | 71 (23.4%) | 46 (15.2%) | 28 (9.2%) | 29 (9.6%) | 19 (6.3%) | 4 (1.3%) | 0 (0.0%) |
| Portugal | High Income | 293 | 59 (20.1%) | 70 (23.9%) | 42 (14.3%) | 63 (21.5%) | 26 (8.9%) | 25 (8.5%) | 4 (1.4%) | 4 (1.4%) |
| Argentina | Upper Middle Income | 282 | 65 (23.0%) | 61 (21.6%) | 57 (20.2%) | 48 (17.0%) | 27 (9.6%) | 20 (7.1%) | 2 (0.7%) | 2 (0.7%) |
| Sweden | High Income | 261 | 37 (14.2%) | 49 (18.8%) | 43 (16.5%) | 66 (25.3%) | 26 (10.0%) | 33 (12.6%) | 3 (1.1%) | 4 (1.5%) |
| Norway | High Income | 256 | 64 (25.0%) | 46 (18.0%) | 41 (16.0%) | 54 (21.1%) | 17 (6.6%) | 32 (12.5%) | 0 (0.0%) | 2 (0.8%) |
| Denmark | High Income | 253 | 40 (15.8%) | 69 (27.3%) | 36 (14.2%) | 67 (26.5%) | 12 (4.7%) | 29 (11.5%) | 0 (0.0%) | 0 (0.0%) |
| Croatia | High Income | 226 | 46 (20.4%) | 47 (20.8%) | 54 (23.9%) | 35 (15.5%) | 30 (13.3%) | 12 (5.3%) | 1 (0.4%) | 1 (0.4%) |
| Hungary | High Income | 220 | 52 (23.6%) | 55 (25.0%) | 33 (15.0%) | 48 (21.8%) | 8 (3.6%) | 23 (10.5%) | 0 (0.0%) | 1 (0.5%) |
| Thailand | Upper Middle Income | 194 | 68 (35.1%) | 28 (14.4%) | 40 (20.6%) | 21 (10.8%) | 21 (10.8%) | 10 (5.2%) | 5 (2.6%) | 1 (0.5%) |
| Romania | High Income | 170 | 41 (24.1%) | 69 (40.6%) | 16 (9.4%) | 28 (16.5%) | 5 (2.9%) | 10 (5.9%) | 0 (0.0%) | 1 (0.6%) |
| Chile | High Income | 164 | 52 (31.7%) | 27 (16.5%) | 34 (20.7%) | 27 (16.5%) | 11 (6.7%) | 9 (5.5%) | 2 (1.2%) | 2 (1.2%) |
| Lebanon | Lower Middle Income | 163 | 64 (39.3%) | 10 (6.1%) | 45 (27.6%) | 14 (8.6%) | 19 (11.7%) | 9 (5.5%) | 2 (1.2%) | 0 (0.0%) |
| Serbia | Upper Middle Income | 154 | 61 (39.6%) | 25 (16.2%) | 27 (17.5%) | 16 (10.4%) | 17 (11.0%) | 6 (3.9%) | 1 (0.6%) | 1 (0.6%) |
| Singapore | High Income | 138 | 38 (27.5%) | 36 (26.1%) | 35 (25.4%) | 16 (11.6%) | 4 (2.9%) | 7 (5.1%) | 2 (1.4%) | 0 (0.0%) |
| Bulgaria | Upper Middle Income | 118 | 27 (22.9%) | 29 (24.6%) | 19 (16.1%) | 34 (28.8%) | 5 (4.2%) | 4 (3.4%) | 0 (0.0%) | 0 (0.0%) |
| New Zealand | High Income | 111 | 32 (28.8%) | 30 (27.0%) | 19 (17.1%) | 18 (16.2%) | 3 (2.7%) | 8 (7.2%) | 0 (0.0%) | 1 (0.9%) |
| Finland | High Income | 108 | 17 (15.7%) | 32 (29.6%) | 15 (13.9%) | 27 (25.0%) | 1 (0.9%) | 16 (14.8%) | 0 (0.0%) | 0 (0.0%) |
| Hong Kong | High Income | 100 | 30 (30.0%) | 34 (34.0%) | 11 (11.0%) | 16 (16.0%) | 3 (3.0%) | 4 (4.0%) | 0 (0.0%) | 2 (2.0%) |
| Slovenia | High Income | 75 | 11 (14.7%) | 23 (30.7%) | 10 (13.3%) | 22 (29.3%) | 1 (1.3%) | 7 (9.3%) | 0 (0.0%) | 1 (1.3%) |
| Egypt | Lower Middle Income | 69 | 40 (58.0%) | 7 (10.1%) | 13 (18.8%) | 5 (7.2%) | 4 (5.8%) | 0 (0.0%) | 0 (0.0%) | 0 (0.0%) |
| South Africa | Upper Middle Income | 53 | 12 (22.6%) | 9 (17.0%) | 11 (20.8%) | 10 (18.9%) | 4 (7.5%) | 7 (13.2%) | 0 (0.0%) | 0 (0.0%) |
| Ukraine | Lower Middle Income | 48 | 20 (41.7%) | 11 (22.9%) | 11 (22.9%) | 6 (12.5%) | 0 (0.0%) | 0 (0.0%) | 0 (0.0%) | 0 (0.0%) |
| Malaysia | Upper Middle Income | 45 | 18 (40.0%) | 6 (13.3%) | 10 (22.2%) | 2 (4.4%) | 5 (11.1%) | 4 (8.9%) | 0 (0.0%) | 0 (0.0%) |
| Vietnam | Lower Middle Income | 40 | 12 (30.0%) | 12 (30.0%) | 5 (12.5%) | 6 (15.0%) | 2 (5.0%) | 3 (7.5%) | 0 (0.0%) | 0 (0.0%) |
| Lithuania | High Income | 37 | 13 (35.1%) | 10 (27.0%) | 2 (5.4%) | 4 (10.8%) | 5 (13.5%) | 3 (8.1%) | 0 (0.0%) | 0 (0.0%) |
| Peru | Upper Middle Income | 37 | 13 (35.1%) | 10 (27.0%) | 3 (8.1%) | 4 (10.8%) | 4 (10.8%) | 2 (5.4%) | 1 (2.7%) | 0 (0.0%) |
| Indonesia | Lower Middle Income | 37 | 21 (56.8%) | 3 (8.1%) | 8 (21.6%) | 0 (0.0%) | 4 (10.8%) | 1 (2.7%) | 0 (0.0%) | 0 (0.0%) |
| Philippines | Lower Middle Income | 35 | 13 (37.1%) | 5 (14.3%) | 9 (25.7%) | 6 (17.1%) | 1 (2.9%) | 1 (2.9%) | 0 (0.0%) | 0 (0.0%) |
| Puerto Rico | High Income | 34 | 6 (17.6%) | 12 (35.3%) | 3 (8.8%) | 4 (11.8%) | 1 (2.9%) | 5 (14.7%) | 1 (2.9%) | 2 (5.9%) |
| Bosnia and Herzegovina | Upper Middle Income | 32 | 17 (53.1%) | 5 (15.6%) | 8 (25.0%) | 1 (3.1%) | 1 (3.1%) | 0 (0.0%) | 0 (0.0%) | 0 (0.0%) |
| Malta | High Income | 27 | 9 (33.3%) | 1 (3.7%) | 9 (33.3%) | 2 (7.4%) | 6 (22.2%) | 0 (0.0%) | 0 (0.0%) | 0 (0.0%) |
| Slovakia | High Income | 25 | 4 (16.0%) | 4 (16.0%) | 8 (32.0%) | 5 (20.0%) | 3 (12.0%) | 1 (4.0%) | 0 (0.0%) | 0 (0.0%) |
| Saudi Arabia | High Income | 22 | 5 (22.7%) | 10 (45.5%) | 3 (13.6%) | 2 (9.1%) | 0 (0.0%) | 1 (4.5%) | 1 (4.5%) | 0 (0.0%) |
| Estonia | High Income | 22 | 0 (0.0%) | 7 (31.8%) | 4 (18.2%) | 4 (18.2%) | 1 (4.5%) | 4 (18.2%) | 2 (9.1%) | 0 (0.0%) |
| Luxembourg | High Income | 21 | 4 (19.0%) | 11 (52.4%) | 3 (14.3%) | 1 (4.8%) | 1 (4.8%) | 1 (4.8%) | 0 (0.0%) | 0 (0.0%) |
| Latvia | High Income | 21 | 7 (33.3%) | 4 (19.0%) | 5 (23.8%) | 1 (4.8%) | 0 (0.0%) | 4 (19.0%) | 0 (0.0%) | 0 (0.0%) |
| UAE | High Income | 16 | 8 (50.0%) | 2 (12.5%) | 5 (31.2%) | 0 (0.0%) | 1 (6.2%) | 0 (0.0%) | 0 (0.0%) | 0 (0.0%) |
| Guatemala | Upper Middle Income | 16 | 3 (18.8%) | 2 (12.5%) | 1 (6.2%) | 3 (18.8%) | 2 (12.5%) | 2 (12.5%) | 2 (12.5%) | 1 (6.2%) |
| Iraq | Upper Middle Income | 16 | 9 (56.2%) | 2 (12.5%) | 3 (18.8%) | 0 (0.0%) | 2 (12.5%) | 0 (0.0%) | 0 (0.0%) | 0 (0.0%) |
| Georgia | Upper Middle Income | 16 | 9 (56.2%) | 4 (25.0%) | 2 (12.5%) | 0 (0.0%) | 1 (6.2%) | 0 (0.0%) | 0 (0.0%) | 0 (0.0%) |
| Costa Rica | Upper Middle Income | 15 | 5 (33.3%) | 3 (20.0%) | 1 (6.7%) | 3 (20.0%) | 1 (6.7%) | 1 (6.7%) | 1 (6.7%) | 0 (0.0%) |
| Iceland | High Income | 14 | 2 (14.3%) | 3 (21.4%) | 1 (7.1%) | 2 (14.3%) | 1 (7.1%) | 4 (28.6%) | 0 (0.0%) | 1 (7.1%) |
| Pakistan | Lower Middle Income | 14 | 8 (57.1%) | 3 (21.4%) | 1 (7.1%) | 0 (0.0%) | 1 (7.1%) | 0 (0.0%) | 1 (7.1%) | 0 (0.0%) |
| Marshall Islands | Upper Middle Income | 13 | 7 (53.8%) | 2 (15.4%) | 3 (23.1%) | 0 (0.0%) | 0 (0.0%) | 1 (7.7%) | 0 (0.0%) | 0 (0.0%) |
| Cyprus | High Income | 13 | 4 (30.8%) | 2 (15.4%) | 4 (30.8%) | 2 (15.4%) | 1 (7.7%) | 0 (0.0%) | 0 (0.0%) | 0 (0.0%) |
| Montenegro | Upper Middle Income | 12 | 6 (50.0%) | 1 (8.3%) | 0 (0.0%) | 0 (0.0%) | 2 (16.7%) | 3 (25.0%) | 0 (0.0%) | 0 (0.0%) |
| Missing | N/A | 7602 | 2031 (26.7%) | 1405 (18.5%) | 1576 (20.7%) | 1149 (15.1%) | 733 (9.6%) | 579 (7.6%) | 77 (1.0%) | 52 (0.7%) |

This classification based on the data from [World Bank](https://data.worldbank.org/country). Countries with more than 10 reports are presented.

# Supplementary Figure F1. The rates of overall and system associated irAEs in 18-64, 65-74, 75-84 and 85-100 age groups.

**
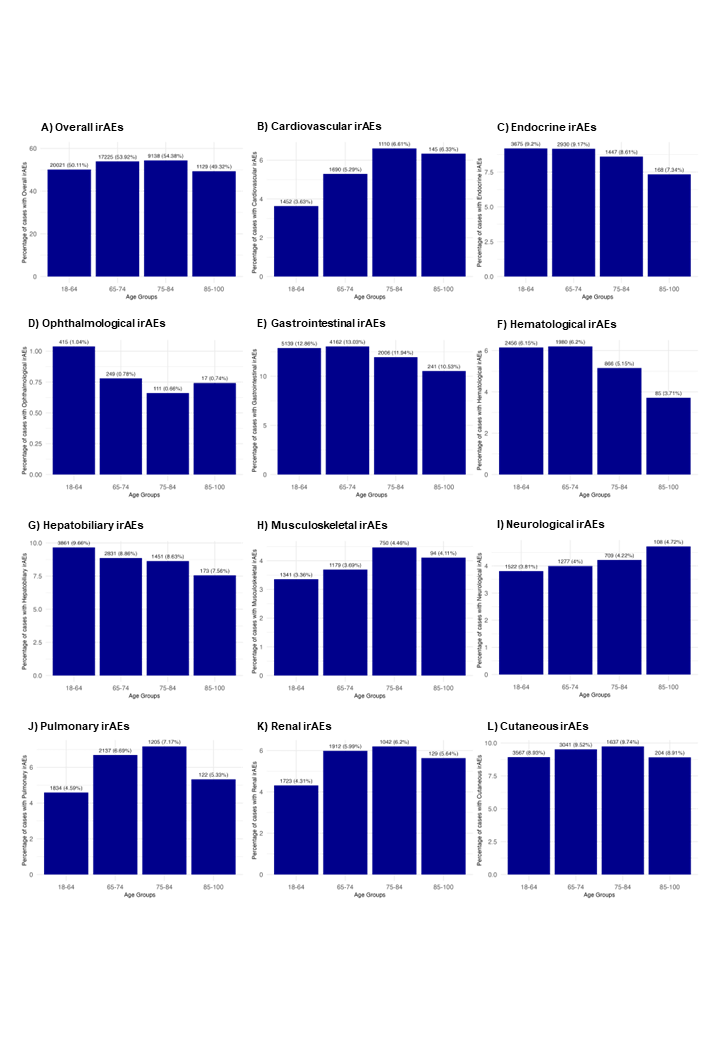
**

# Supplementary Table S5. Univariate Analysis of Factors Influencing Immune-Related Adverse Events

| **Variable** | **Odds Ratio** | **Confidence Intervals** | **p_value** | **Significance** | **Estimate** | **Std. Error** | **z_value** |
| --- | --- | --- | --- | --- | --- | --- | --- |
| *(Intercept)* | 33.363 | (2.9799 - 3.7363) | < 2.2e-16 | *** | 12.049 | 0.0577 | 208.789 |
| *Sex* | 1.026 | (0.9951 - 1.0578) | 0.0998444 | . | 0.0257 | 0.0156 | 16.456 |
| *Additional Targeted Therapy* | 12.829 | (1.2223 - 1.3466) | < 2.2e-16 | *** | 0.2491 | 0.0247 | 100.819 |
| *Additional Chemoherapy* | 13.292 | (1.272 - 1.3891) | < 2.2e-16 | *** | 0.2845 | 0.0225 | 126.666 |
| *Vivolumab* | 0.8041 | (0.7414 - 0.8719) | 1,34E-04 | *** | -0.218 | 0.0413 | -52.737 |
| *Pembrolizumab* | 11.737 | (1.0757 - 1.2805) | 0.0003153 | *** | 0.1602 | 0.0445 | 36.024 |
| *Cemiplimab* | 0.889 | (0.6861 - 1.1546) | 0.3750392 |  | -0.1177 | 0.1327 | -0.8871 |
| *Dostarlimab* | 0.4432 | (0.2867 - 0.6769) | 0.0001948 | *** | -0.8138 | 0.2184 | -37.256 |
| *Atezolizumab* | 0.7379 | (0.6699 - 0.8126) | 6,77E-07 | *** | -0.3039 | 0.0493 | -61.714 |
| *Durvalumab* | 0.7027 | (0.6229 - 0.7926) | 9,41E-06 | *** | -0.3528 | 0.0615 | -5.741 |
| *Avelumab* | 0.586 | (0.499 - 0.6877) | 6,51E-08 | *** | -0.5344 | 0.0818 | -65.316 |
| *Ipilimumab* | 16.075 | (1.5431 - 1.6748) | < 2.2e-16 | *** | 0.4747 | 0.0209 | 227.216 |
| *Tremelimumab* | 14.839 | (1.2469 - 1.7672) | 9,14E-03 | *** | 0.3947 | 0.089 | 44.366 |
| *Relatlimab* | 0.6457 | (0.5044 - 0.8242) | 0.0004718 | *** | -0.4375 | 0.1251 | -34.963 |
| *Healthcare professional* | 0.6236 | (0.6 - 0.6482) | < 2.2e-16 | *** | -0.4722 | 0.0197 | -239.372 |
| *Report Year* | 10.568 | (1.0493 - 1.0644) | < 2.2e-16 | *** | 0.0553 | 0.0037 | 150.848 |
| *The economic status of the reporting country* | 0.5446 | (0.5202 - 0.5701) | < 2.2e-16 | *** | -0.6076 | 0.0233 | -260.316 |
| *Indication* | 0.9767 | (0.9738 - 0.9796) | < 2.2e-16 | *** | -0.0236 | 0.0015 | -155.477 |

Significance codes: 0 '***' 0.001 '**' 0.01 '*' 0.05 '.' 0.1 ' ' 1
